# Axial variation in flexural stiffness of plant stem segments: measurement methods and the influence of measurement uncertainty

**DOI:** 10.1186/s13007-021-00793-8

**Published:** 2021-10-07

**Authors:** Nathanael Martin-Nelson, Brandon Sutherland, Michael Yancey, Chung Shan Liao, Christopher J. Stubbs, Douglas D. Cook

**Affiliations:** 1 Department of Mechanical Engineering, Brigham Young University, Engineering Building 350, Provo, UT 84602 USA; 2 School of Computer Sciences and Engineering , Fairleigh Dickinson University , 1000 River Rd , Teaneck , NJ 07666 USA

## Abstract

**Background:**

Flexural three-point bending tests are useful for characterizing the mechanical properties of plant stems. These tests can be performed with minimal sample preparation, thus allowing tests to be performed relatively quickly. The best-practice for such tests involves long spans with supports and load placed at nodes. This approach typically provides only one flexural stiffness measurement per specimen. However, by combining flexural tests with analytic equations, it is possible to solve for the mechanical characteristics of individual stem segments.

**Results:**

A method is presented for using flexural tests to obtain estimates of flexural stiffness of individual segments. This method pairs physical test data with analytic models to obtain a system of equations. The solution of this system of equations provides values of flexural stiffness for individual stalk segments. Uncertainty in the solved values for flexural stiffness were found to be strongly dependent upon measurement errors. Row-wise scaling of the system of equations reduced the influence of measurement error. Of many possible test combinations, the most advantageous set of tests for performing these measurements were identified. Relationships between measurement uncertainty and solution uncertainty were provided for two different testing methods.

**Conclusions:**

The methods presented in this paper can be used to measure the axial variation in flexural stiffness of plant stem segments. However, care must be taken to account for the influence of measurement error as the individual segment method amplifies measurement error. An alternative method involving aggregate flexural stiffness values does not amplify measurement error, but provides lower spatial resolution.

## Introduction

Three-point bending tests are frequently used to characterize the mechanical properties of plant stems [1–3]. In contrast to other methods, such as compression or tensile testing, this testing approach requires minimal sample preparation, allowing tests to be performed relatively quickly [4, 5]. Two types of tests can be performed using three-point bending: non-destructive flexural tests and destructive bending strength tests [3]. Flexural stiffness can be obtained from both tests, and has been shown to be highly correlated with bending strength (*ibid*). Flexural stiffness measurements can also be used to obtain mechanical tissue properties information without damaging or dissecting the specimen [5, 6]. Flexural stiffness also provides information regarding gradients in morphology and material properties which are closely related to mechanical stability [6, 7].

**Fig. 1:**
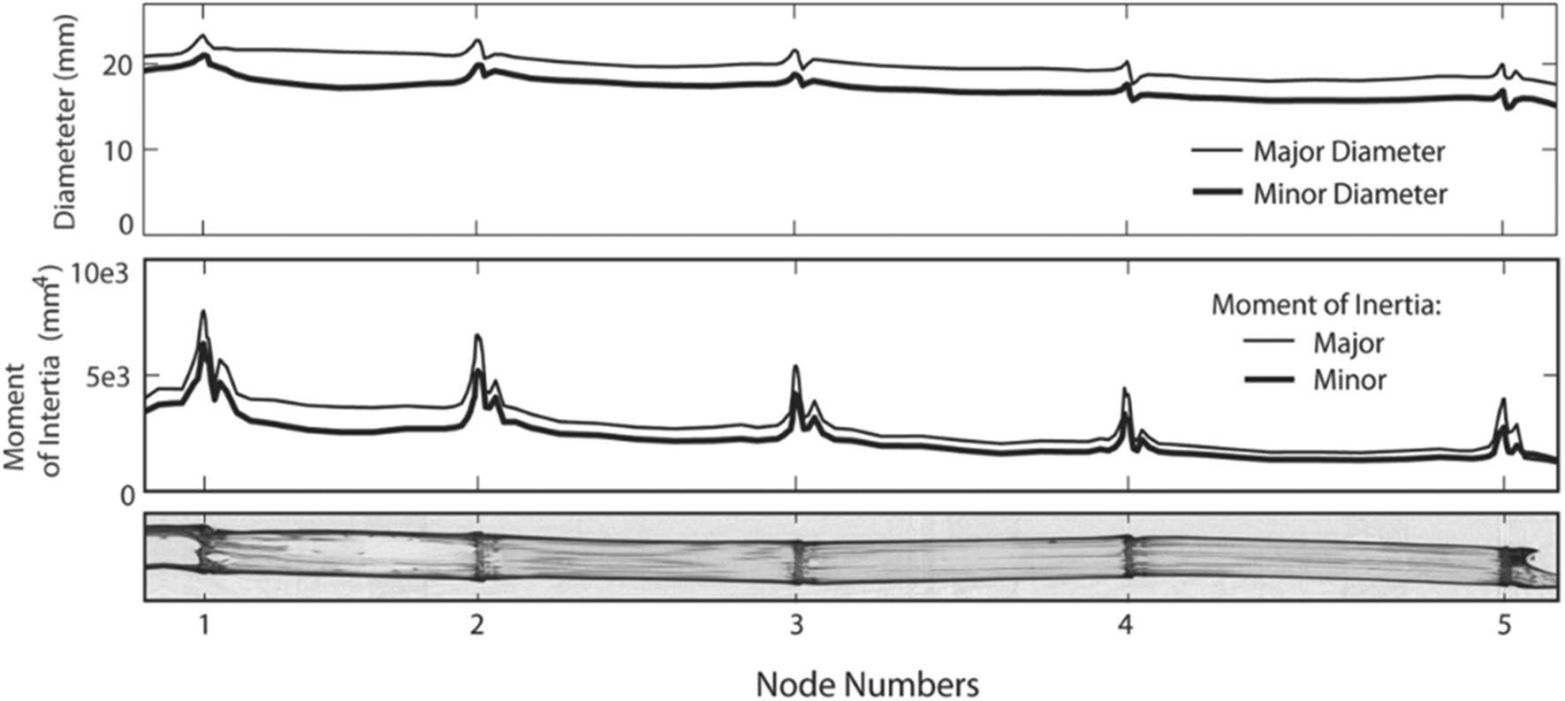
Major and minor diameter of a maize stalk cross-section along the length of the stalk. Bottom image is from a CT scan where darkness of the tissue is proportional to tissue density (image from [10], used with permission)

Flexural tests of plant stems typically exhibit good repeatability values [5]. Plant stems can be tested in three-point bending as long as care is taken to minimize the influence of transverse compression of the stem cross-section, which can introduce serious errors [8, 9]. This implies that loads should be placed only at the junction between segments, which are known as nodes. Figure 1 shows a CT scan cross-section of a maize stalk (adapted from a prior study [10]). The nodes can be seen as dark regions between segments. The higher density of nodal regions makes them far less susceptible to transverse compression than internodes (the regions between nodes).

**Fig. 2:**
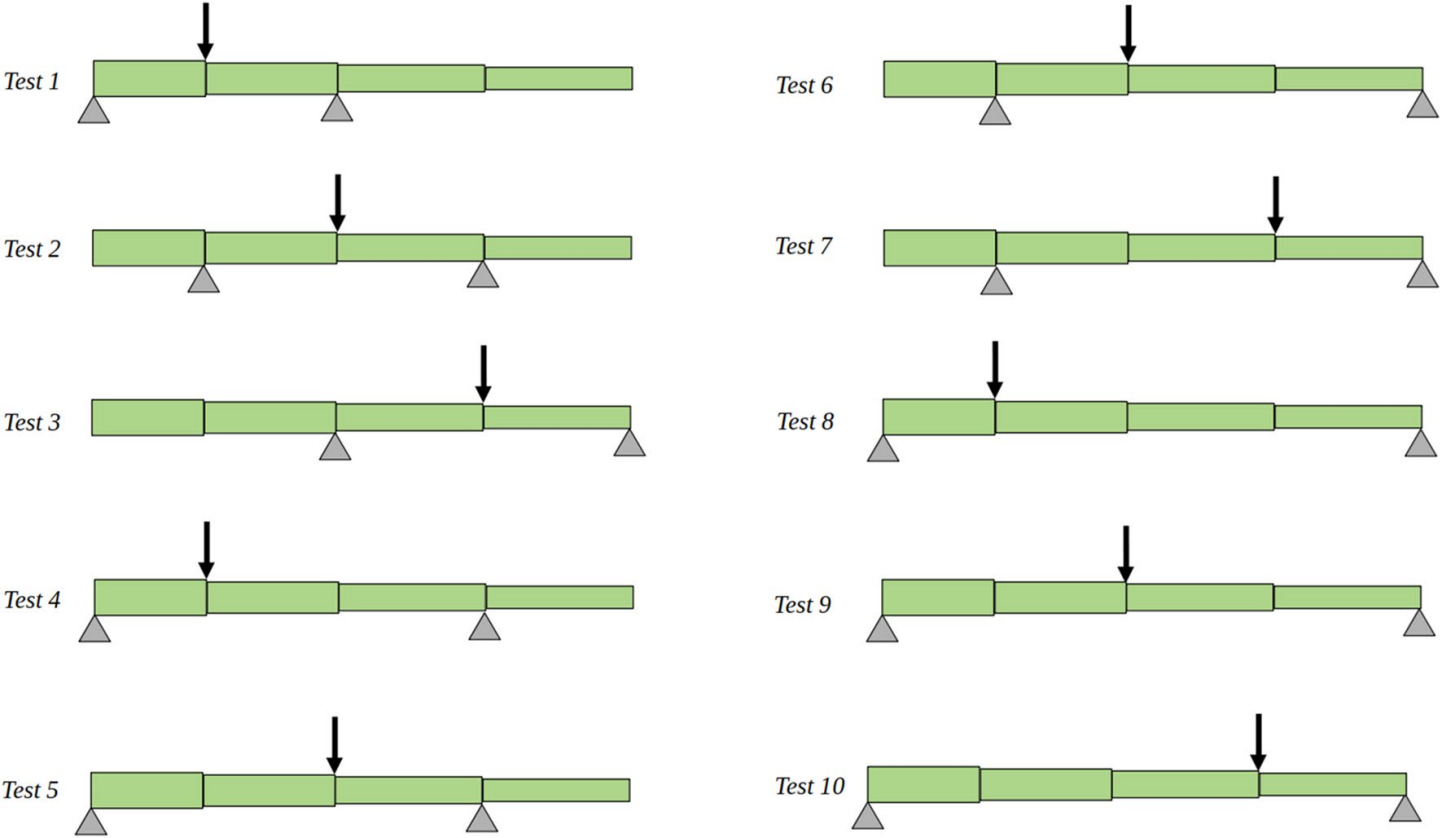
The 10 possible testing configurations for a stem specimen consisting of four internodes and 5 nodes

Because loads and supports must be placed at nodal locations [8, 9], the shortest test that can be performed includes three nodes and two internodes (Tests 1–3 in Fig. 2). Three-point bending tests can be expanded to include longer spans as well as asymmetric tests (Fig. 2, Tests 3–10).

Each of the tests shown in Fig. 2 involves multiple segments. This study focuses on the idea that multiple tests can be combined with analytic equations to solve for the flexural stiffness of each individual internodal section. This would enable future researchers to measure the variation of flexural stiffness and mechanical tissue properties along the length of plant stems without inducing damage to the specimen. This information would provide valuable insights into structural patterns and to open questions such as the optimal tapering of plant stems [7]. However, a major challenge in this approach is the influence of experimental error on the solution, which can present a challenge to the accuracy of biomechanical models [11–13].

The purpose of this study was to develop the equations needed to solve for the flexural stiffness of individual stem segments and quantify the influence of experimental errors on the solution process. This study also addresses the practical feasibility and limitations of this method and provides practical recommendations for addressing measurement error in such tests. This study focuses on maize stalks because of their high economic importance [10, 14, 15].

## Methods

This study utilized experimental, analytical, and computational methods. Analytical beam models were used to formulate the system of linear equations required to solve for the flexural stiffness of individual internodes. The influence of measurement uncertainty on solution uncertainty was explored using an empirical/synthetic data set. Throughout the paper, uncertainty is quantified in terms of the “standard measurement uncertainty” (*u*, standard deviation of error values) [16]. This allows the reader to readily convert uncertainty into small sample or large sample coverage intervals as needed [17].

### System of equations and modeling assumptions

Like many plants, maize stalks are characterized by periodic nodes and internodes (see Fig. 1). The internodal region is fairly uniform in cross-sectional shape [11]. From the base to apex, each successive internode is slightly smaller than the one preceding it (see Fig. 1). Maize stalks are therefore commonly approximated as a series of segments, each of which has a constant cross-section and constant material properties [8]. In this study, we assume and solve for a constant aggregate flexural stiffness for each internode. Stubbs et al. demonstrated that the Euler–Bernoulli beam theory is relevant to plant stems even though plant tissues are neither isotropic nor homogeneous in the cross-section [18].

**Fig. 3:**
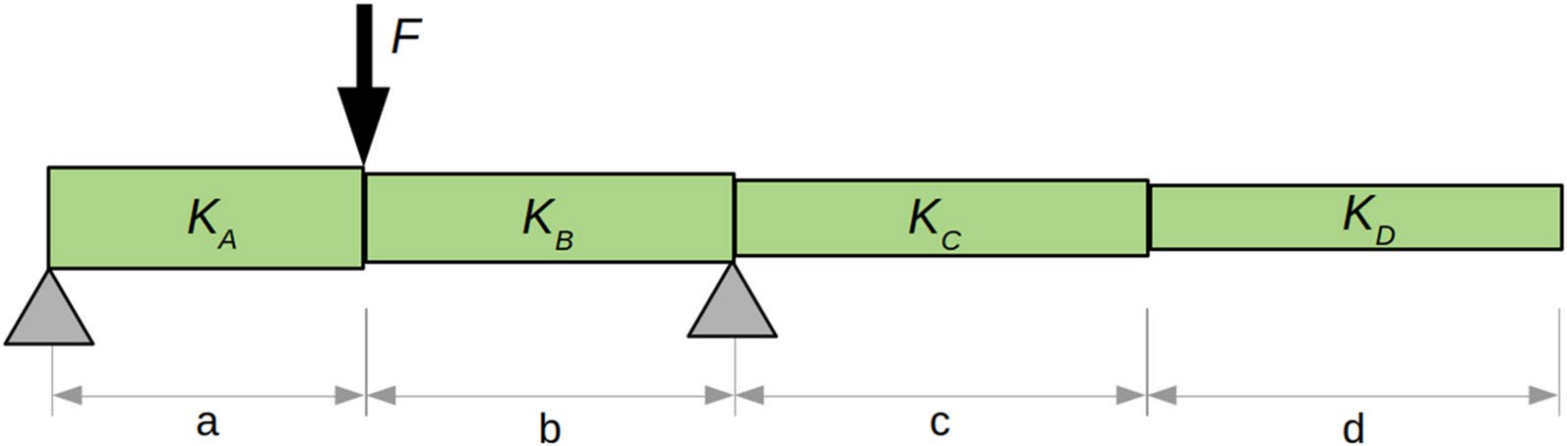
Diagram of a 4-internode stem in the configuration of Test #1. Labels indicate the length of each internode (a–d) as well as the flexural stiffness of each internode (*K*_*A*_*—K*_*D*_)

Briefly stated, Euler–Bernoulli beam theory and Castigliano’s Theorem were used to write equations relating displacement and flexural stiffness for each of the testing configurations shown in Fig. 2. Flexural stiffness is given the symbol *K*, and each internode is designated by a letter A–D (*K*_*A*_, *K*_*B*_, *K*_*C*_, *K*_*D*_). Finally, the length of each stem segment was designated by the corresponding lower case letter (a–d). A diagram illustrating these quantities, as well as the test configuration and the corresponding force/displacement equation for Test 1 are provided in Fig. 3.

1 $$\frac \delta {F}={3(a+b)^2}\left ( \frac{a^3b^2}{I_{EA}}+ \frac{a^2b^3}{I_{EB}}\right).$$

The reader can confirm in a few steps of algebra that Eq. 1 reduces to the familiar equation for symmetric three-point bending of a uniform beam (δ = FL^3^/(48EI)) when both a and b have values of L/2, and when *K*_*A*_ = *K*_***B***_ = *EI*. The full set of 10 equations is shown below. In this equation, *ɸ*_*i*_ indicates the force/displacement slope of each test.2

### Aggregate stiffnesses

An alternative to the system of equations shown above is to only utilize tests 1, 2, and 3. Then, instead of solving for the 4 individual flexural stiffness values (*K*_*A*_, *K*_*B*_, *K*_*C*_, *K*_*D*_), three *aggregate* flexural stiffness values are obtained (*K*_*AB*_, *K*_*BC*_, *K*_*CD*_). This approach is similar to Eq. 1, but a single aggregate flexural stiffness value is assumed for the entire two-internode span. For example, Test 1 can be used with the following equation to obtain the aggregate stiffness *K*_*AB*_:

3 $$K_{AB} ~=~ \frac{F}{\delta} \frac{a^2 b^2}{3(a+b)}.$$

This approach requires only three tests, but does not provide the spatial resolution of the system of equations shown above.

### Validation of analytic equations

The analytic system of equations described above was validated by creating a model stalk and simulating the set of 10 tests using commercial finite-element software (FEA). Close agreement between the analytic model and the finite-element models was found. The maximum discrepancy between the finite element model and the analytic equations was 2.2%, which was a result of the finite element models accounting for shear deformation whereas the analytic equations do not. The issue of shear deformation is discussed further in “Limitations” section.

### Uncertainty analysis

All four unknown *K* values can be obtained by choosing any combination of 4 or more tests. A combinatorial analysis revealed that there are 848 different test combinations that can be used to solve for the unknown *K* values (e.g., 10 choose 4 = 210 test combinations; 10 choose 5 = 252 combinations;… etc.). Unfortunately, most of these test combinations are ill-conditioned. Even when all 10 tests are used, the solution process amplifies measurement errors, leading to unreliable solution values. A series of analyses were therefore performed to determine (a) if all combinations of these equations were equally prone to this effect and (b) the sensitivity of the system to errors in the measurement of length and force/displacement slope.

The investigation of these issues was performed with a combination of experimental and synthetic data. Experimental data on stalk morphology was incorporated to accurately represent real plant specimens. By augmenting experimental data with synthetic data, it was possible to *pre-specify* the values of experimental measurement errors. This in turn allowed us to determine the magnitude of resulting solution errors.

#### Experimental/synthetic test data

Experimental data consisted of internodal lengths, internodal diameters, and rind thickness values from 200 maize stalks (data graciously provided by Dr. Daniel Robertson, University of Idaho). The area moment of inertia of each internode was computed using the elliptical cross-section assumption [19]. Finally, flexural stiffness values for each internode were calculated by multiplying the moment of inertia by a longitudinal modulus of elasticity value. Modulus of elasticity values were not varied within each stalk since little is currently known about the pattern of variation of this factor within individual stalks. The modulus of elasticity for each stalk was randomly selected from within a normal distribution with mean value of 11.1 GPa and a standard deviation of 2 GPa. This distribution was chosen because it spans the ranges of modulus values reported previously with validation across multiple measurement modalities [4, 5]. Lengths and flexural stiffness values were entered into the analytic equations to compute a corresponding force/displacement slopes. The resulting set of length, flexural stiffness, and force/displacement values were assumed to have zero error and were used as reference data throughout the study. Summary statistics for the zero-error data set is shown in Table 1.

**Table 1:** Summary statistics for the data used for analysis of solution methods

	Length (mm)		Major diameter (mm)		Minor diameter (mm)		Rind thickness (mm)		Moment of inertia (mm^4^)		Modulus of elasticity (Gpa)		Flexural stiffness (Nm^2^)	
Internode A	153.7	22.9	19.3	2.8	17.0	2.5	1.7	0.5	1699.3	972.5	11.1	2.0	18.8	11.2
Internode B	175.1	23.5	18.0	2.9	16.0	2.4	1.5	0.4	1244.2	713.4			13.7	8.1
Internode C	173.5	24.2	16.6	2.9	15.1	2.3	1.3	0.5	1313.6	765.9			14.5	8.8
Internode D	187.4	24.2	14.8	2.9	14.3	2.4	1.2	0.6	980.4	579.2			10.8	6.7
Type	Measured		Measured		Measured		Measured		Calculated		Sampled		Calculated	

To simulate the physical testing process, random errors were added to each internode length and each force/displacement slope to create synthetic test data. This approach allowed us to control the degree of measurement uncertainty in each measurement type. The equations for generating random errors are shown below (for the sake of brevity, we show equations for only a single internode and a single force/displacement slope):

4 $$a_{i} = a_{0} + \epsilon_i        \epsilon_i \sim N(0,u_L),$$

5 $$\phi_i =  \phi_0 + \epsilon_i        \epsilon_i \sim N(0,u_{\phi}).$$

In these equations, *a*_*i*_ represents a realistic value for the measurement of the length of segment A. The measurement process is simulated by modifying the true length, a_0_ by the introduction of error *ϵ*_*i*_. Similarly, the quantity *ɸ*_*i*_ represents a realistic measurement which has its reference value (*ɸ*_0_) modified by a random measurement error. All error values were drawn from a Gaussian (normal) distribution with a mean of zero and a specified uncertainty for length (*u*_*L*_) or slope (*u*_*ɸ*_). Throughout this paper, uncertainties are specified as standard measurement uncertainties (i.e., standard deviation of measurement errors) [17].

#### Experimental design

A factorial design was used with three levels for uncertainty in length (0, 1, and 2 mm), three levels of slope uncertainty (0, 2.5%, and 5%), 200 different stalks, and the 848 possible test combinations. At each design point, a set of 50 realistic measurements were created using the method outlined in Eqs. 3 and 4. Each replicate data set thus represented a potential set of real measurement data. This sampling scheme resulted in ~ 50 million simulated solution processes. This large number was possible because the analytic models could be created and solved in a small fraction of a second. For each simulated solution process, the primary quantity of interest was the 4 relative errors (one for each flexural stiffness). Additional quantities were also calculated, including the condition number of the original system and the condition number of the system after scaling such that each row of the system had a maximum value of unity.

## Results

### Error distributions

Simulated measurement errors at various levels caused corresponding errors in *K* values. We first examine the situation in which all 10 tests/equations were used. The standard uncertainty in length measurements was set at 1 mm and the uncertainty in slope measurement was set at 2.5%. These values were used to simulate the 10-test procedure for 50 different stalks. This process was repeated 50 times, thus providing a total of 2500 simulated solutions for each internode. In parallel with these simulations, Tests 1, 2, and 3 were used to solve for the aggregate flexural stiffnesses corresponding to each test.

**Fig. 4:**
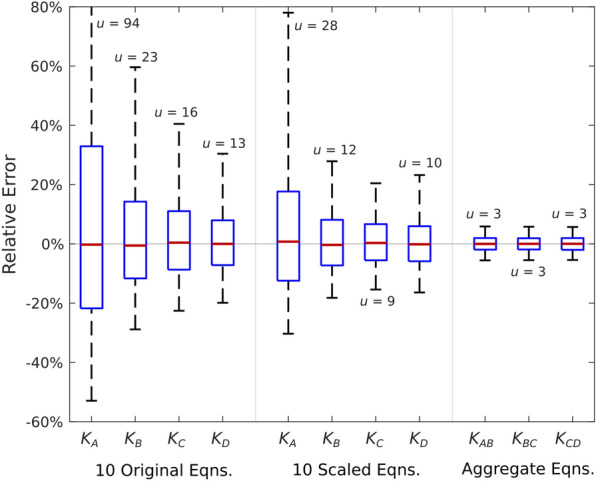
Distribution of errors across stalks and replicates when using all 10 equations. Each box summarizes 2500 relative error values. Boxes indicate the 25th, 50th, and 75th percentiles while the whiskers span 95% of the data. Standard uncertainty values (*u*) are provided for each distribution

Simulated solution values were then compared to the true values. The distributions of the relative error for these solutions were used to describe the solution uncertainty, as seen in Fig. 4. As expected, error bias (the horizontal red bars in each box of Fig. 4) was relatively low, indicating that each solution method was unbiased. When solving for individual internode stiffness values, scaled equations reduced the uncertainty of each distribution, and are therefore used in the presentation of subsequent results. The aggregate method exhibited the lowest uncertainty of the three solution methods.

**Fig. 5:**
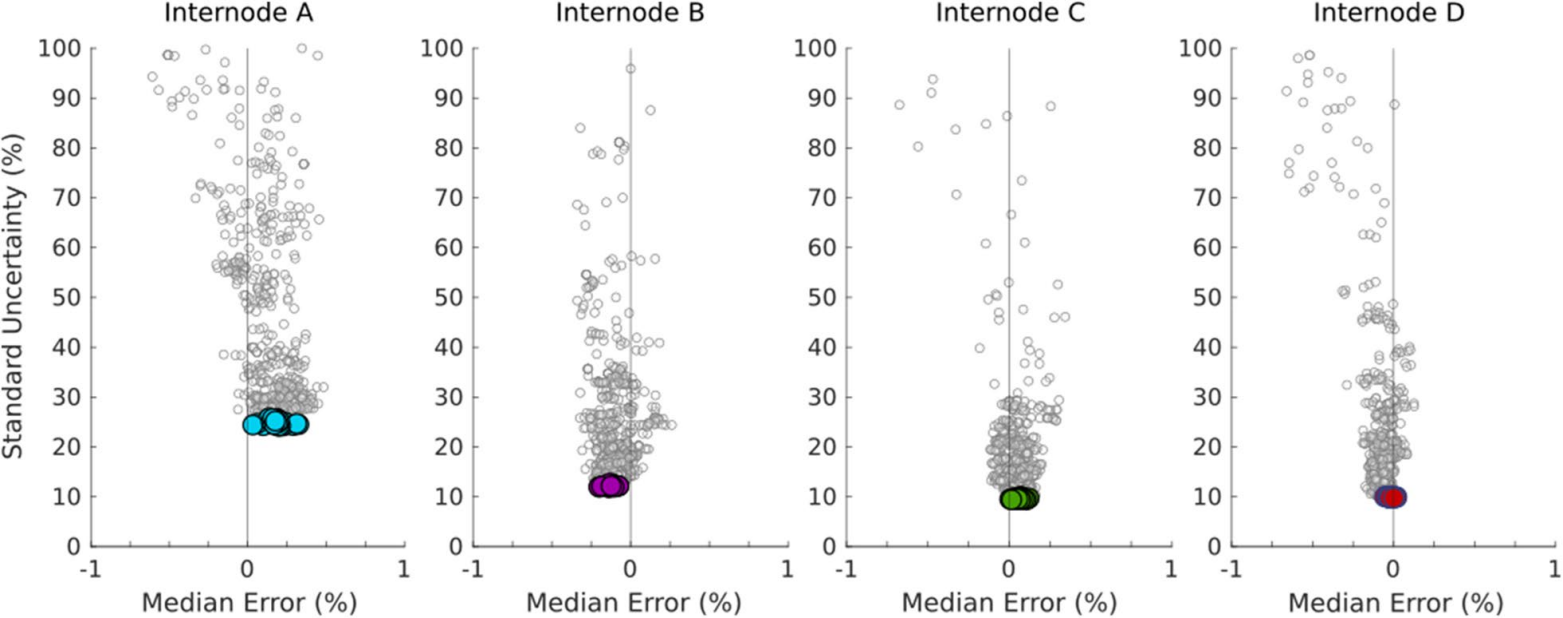
Scatter plots for results from each internode showing the median error and standard uncertainty of various test combinations. The 32 best test combinations are shown by larger, outlined dots. Data in this figure corresponds to systems with standard uncertainty values of ± 1 mm on length measurements, ± 2.5% on slope measurements, and in which all rows were normalized before solving the system of equations

The error distributions for each of the 848 test combinations were similarly characterized by bias (the median error) and standard uncertainty (*u*). Across the 848 test combinations, bias values were clustered around zero, with over 99% of test combinations exhibiting bias values less than 1%. While bias was consistently low, the spread of error values varied widely. Figure 5 depicts bias/uncertainty scatter plots in which each dot represents the error distribution of a single test combination from among the 848 possible test combinations. As seen in this figure, uncertainty values ranged from approximately 10% to over 100%. Some test combinations even had uncertainty values as high as 1000%. This scatter plot of Fig. 5 has been scaled to show the most relevant test combinations (i.e. unusually high values of bias and spread are not shown).

Figure 5 also highlights the “best combinations”. These test combinations were found to have the lowest uncertainty among all possible test combinations. Furthermore, these test combinations were consistent across all 4 internodes. For internodes B–D, the best combinations exhibited median errors less than 0.25% and uncertainty levels of less than 13%. The uncertainty for internode A was consistently higher, with levels in the neighborhood of 25%. The reasons for this are not yet fully understood, but errors seem to be roughly proportional to the overall stiffness (compare the uncertainty levels in Fig. 5 to the stiffness values listed in Table 1).

### Best combinations, condition number, and scaling

**Fig. 6:**
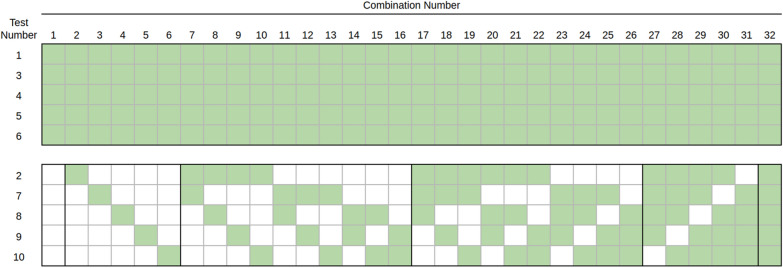
The 32 best test combinations. Each column represents one test combination where green indicates that a test was included and white indicates that a test was omitted. As seen in this figure, tests 1, 3, 4, 5, and 6 were universally included in the set of 32 best test combinations along with any combination of tests 2, 7, 8, 9 and 10

A set of 32 test combinations was found to have lower uncertainty than the remaining tests. These 32 test combinations had similar bias and uncertainty characteristics. An examination of these 32 tests revealed that tests 1, 3, 4, 5, and 6 were present in each of the 32 best test combinations. There appeared to be little or no dependence upon the remaining 5 tests as the full set of 32 consisted of Tests 1, 3, 4, 5, and 6 plus all possible combinations of tests 2, 7, 8, 9, and 10. These combinations are shown graphically in Fig. 6.

The 32 “best” test combinations exhibited generally lower condition numbers than the other test combinations. However, the condition number was not reliably predictive of error levels. This is because the condition number is a holistic matrix-level and vector-level inequality [21]. As such, while it gives a general indication of whether or not a system is ill-conditioned, it does not predict individual errors. In order to obtain accurate estimates of uncertainties, a direct sampling approach like the one used in this study is required.

Across test combinations, row-wise scaling was generally found to reduce error levels, though the effect was inconsistent. For example, row-wise scaling reduced the solution uncertainty by moderate amounts for 16 of the 32 best combinations (percent reduction ranging from 4 to 23%). For 8 test combinations, scaling had a larger effect, with solution uncertainty being reduced by 34–58%. Each of these 8 test combinations included test #10. Finally, for 8 test combinations, scaling provided a very substantial reduction, with reduction in uncertainty ranging from 73 to 82%. Each of these test combinations included Tests #9 and #10. Row-wise scaling improves these systems because it eliminates the disparity in force/displacement slopes (tests with longer spans have much lower force/deformation values than tests with shorter spans).

### Solution uncertainty as a function of measurement uncertainties

**Fig. 7:**
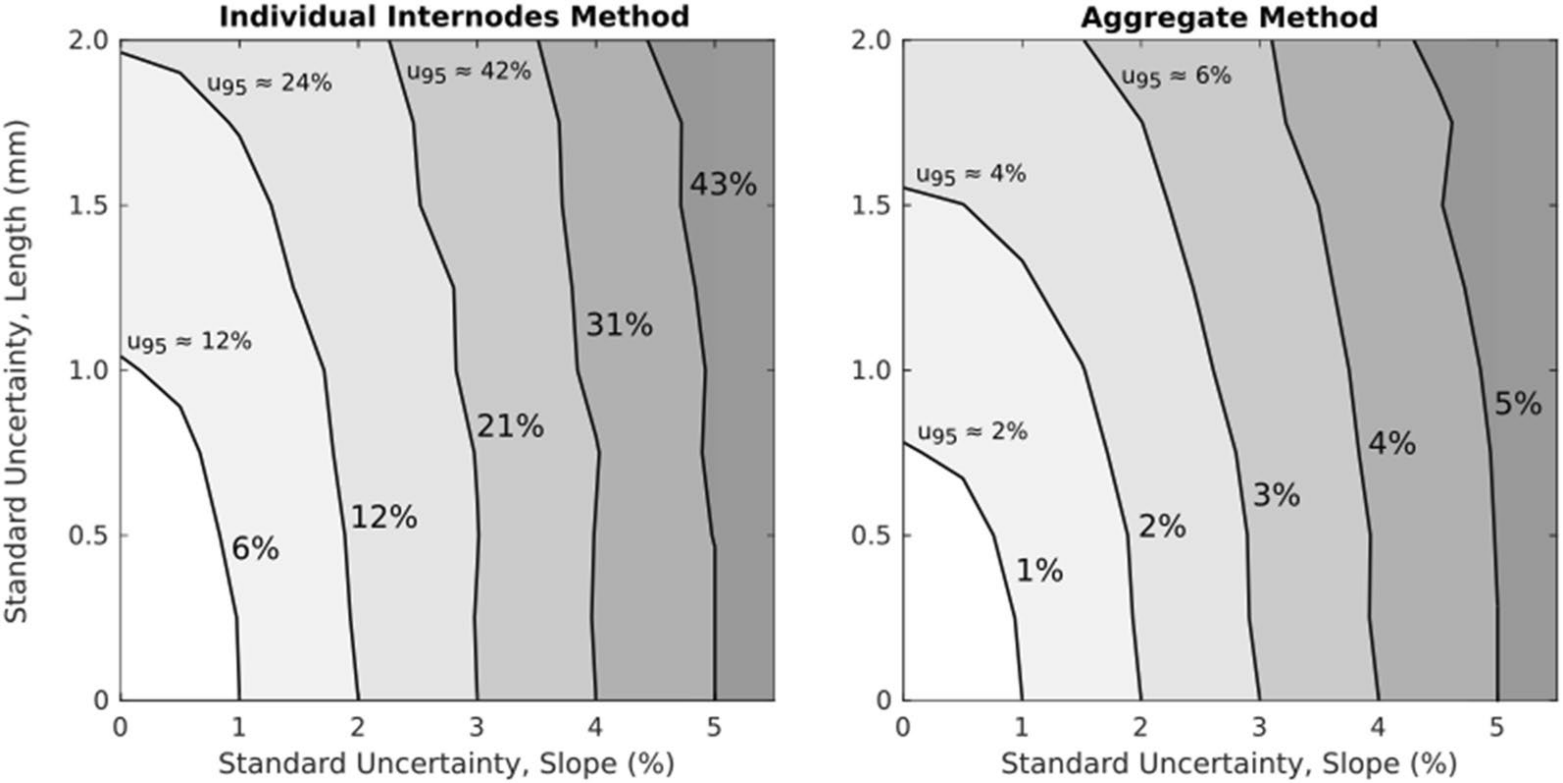
Contour plots depicting the standard uncertainty (standard deviation) in solved values as functions of uncertainty in slope and length measurements. Note that slope errors are specified in percentage while length error is specified in mm. The half-width of 95% confidence interval widths (*u*_95_) are given for several contours as an additional point of reference

The relationships between input uncertainties and the resulting output uncertainty are most readily visualized using contour plots. Two contour plots were created: one for the individual internodes stiffness method and one for the aggregate stiffness method. This was done by pooling together 2500 error values from each of the four internodes for a total of 10 k error values at each sample point. The standard uncertainty of the pooled data was then computed. Contour plots are shown in Fig. 7 below. Note that the axes of each plot are in terms of standard uncertainty, but specified in different units.

Because the best test combinations exhibited virtually identical uncertainty characteristics (see Fig. 5), the left-hand panel of Fig. 7 is representative of the entire group of 32 best test combinations. Differences in uncertainty between the individual and aggregate solution methods can be seen by comparing the isolines between the two contour plots. For example, the first uncertainty isoline (6%) in the left-hand panel of Fig. 7 intersects both axes at approximately 1%. This implies that a 1% measurement uncertainty is amplified by this method, thus producing a solution uncertainty of approximately 6%. This is due to the ill-conditioned nature of the individual internodes method. In contrast, the first uncertainty isoline for the aggregate method (1%) intersects each axis at or below 1%. This indicates that this method does not amplify measurement error.

## Discussion

We have shown that multiple tests can be used to solve for the stiffnesses of individual segments of a plant stem. However, in applying this method to a representative set of test data, we discovered that the system of equations was ill-conditioned. In practical terms, this means that measurement errors are amplified by the solution process. For the maize stalks used in this study, the amplification factor ranged between 6 and 8. The aggregate solution method did not amplify measurement errors. The disadvantage of the aggregate method is that it provides lower spatial resolution than the individual segment method.

### Practical applications

Several practical issues should be considered when applying either of these methods. The first consideration is to quantify the measurement uncertainty of one’s test equipment. Manual measurement of plant stems with a ruler or tape measure yields standard uncertainty values of around 1 mm (equal to 95% uncertainty of about ± 2 mm). Reliability of slope measurements will depend upon the equipment. Levels of slope uncertainty of 1% have been reported [6], but levels of 2.5% may be more representative for most testing arrangements. Uncertainty can be ascertained by performing repeated tests with a small number of specimens. If the equipment is properly calibrated, the standard deviation of repeated tests provides a reasonable estimation of measurement uncertainty [22]. Values for measurement error can then be used in conjunction with Fig. 7 to estimate the resulting solution uncertainty.

Internodal patterns should also be considered. The morphology of most segmented plant stems suggests that *K*_*A*_ > *K*_*B*_ > *K*_*C*_ > *K*_*D*_. The morphological data of Table 1 shows that flexural stiffness of maize internodes decreases by approximately 20–30% between each pair of internodes. Sufficiently high levels of uncertainty could cause this stiffness pattern to be obscured or reversed between some pairs of internodes. For example, let us assume a conservative estimate for the change between segments of 20%. In this situation, the standard uncertainty of the solution would need to be less than 5% to minimize the possibility in which *K*_*A*_ is found to be less than *K*_*B*_ even though the reality is that *K*_*A*_ is greater than *K*_*B*_. As seen in Fig. 7, a solution uncertainty of less than 5% would require relatively low measurement uncertainties.

Another consideration is which tests to perform. The results of this analysis suggest that any of the 32 test combinations shown in Fig. 6 can be used, and that all these combinations produce similar error profiles. One’s intuition may suggest that performing all 10 tests would provide an advantage by means of error cancellation (due to the use of a least-squares solution). However, in our simulations, the inclusion of 10 tests provided no additional benefit to performing any tests beyond tests 1, 3, 4, 5, and 6.

One may consider performing Tests 1–6. This set of tests provides the ability to solve for both aggregate stiffnesses (using Tests 1–3), and/or individual internodal stiffnesses (Tests 1–6). Of course, if total testing time is to be reduced, the fastest approach is to use Tests 1–3 and solve for aggregate stiffness values only.

We also examined the possibility of performing all 10 tests, and then using the resulting data to solve the system repeatedly (i.e., 32 solutions, one for each test combination in Fig. 6). The idea here was that these 32 solutions could then be averaged to further reduce the solution error. Unfortunately, this approach did not provide any additional accuracy. This is because each set of 10 physical tests (see Fig. 2) has a unique measurement error profile. Each measurement error profile produces a corresponding solution error profile. The solution error profile is roughly the same for each of the 32 solution methods, which is why averaging does not improve the accuracy of the result.

There is one final alternative if individual stiffness values are desired but measurement uncertainty cannot be reduced. This is to perform multiple replicates of tests 1, 3, 4, 5, and 6 on each specimen. Average values for internode length and force/displacement slope would then be used in the solution process. This approach will reduce the overall measurement uncertainty and can thus be used to reduce the uncertainty in computed outcomes. Assuming a small sample size (t-distribution), repeating each test six times is sufficient to obtain a mean value for each test that has half the measurement uncertainty of a single test.

### Case study

**Fig. 8:**
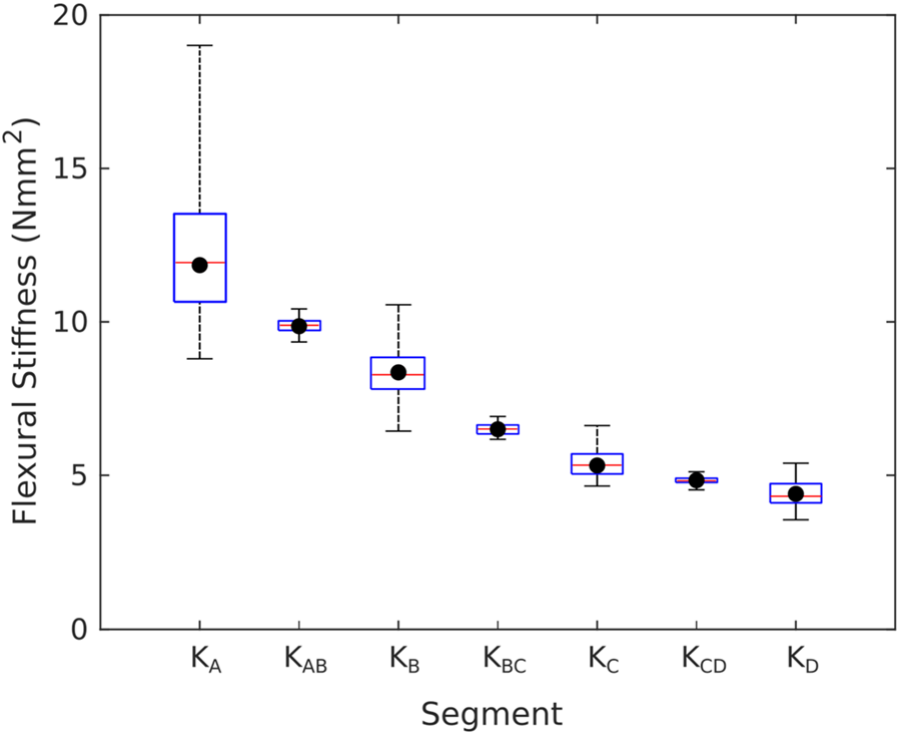
A realistic data set consisting of 50 replicates of synthesized measurements performed on a single stalk with standard uncertainty for length and slope of 1 mm and 2.5%, respectively. Circles represent the exact values for each stiffness value. Boxes indicate the distributions of solved values. Each box represents the 25th, 50th, and 75th percentile. Whiskers span 95% of the distribution. Smaller boxes indicate the aggregate flexural stiffnesses obtained for adjacent internodes by using Tests 1–3 (see Fig. 2 for test numbers)

To illustrate practical outcomes, a realistic scenario was simulated. In this scenario, the set of standard uncertainty levels for length and slope measurements were set at 1 mm and 2.5%. With these levels of uncertainty, the average uncertainty in flexural stiffness measurements (Fig. 7) should be approximately ± 17% (95% uncertainty is approximately ± 34%). A set of 50 instances of this case were simulated for a single stalk. The distribution of solutions and true flexural stiffness values (red circles) are shown below in Fig. 8. As expected, the median value of each distribution lies very close to the true value. However, the uncertainty ranges for each internode are highly variable, with the first internode exhibiting whiskers ranging from 57% above the true value to 35% below the true value while the last internode has a 95% uncertainty of less than 20%. Thus, the average measurement uncertainty can give rise to relatively high values of solution uncertainty due to the nature of the system.

The aggregate flexural stiffness approach is also illustrated in Fig. 8, depicted by the smaller boxes that lie between the larger boxes. Even with the same levels of uncertainty in length and slope measurements, the aggregate approach provides much narrower solution uncertainties. This approach also requires the fewest number of tests. The only disadvantage to this approach is that it does not provide individual flexural stiffness values. It should be noted that the aggregate value is an aggregate of internodes A and B while the second value is a aggregate of internodes B and C. Nevertheless, a comparison between the aggregate results and the actual stiffness values shown in Fig. 8 demonstrate that this approach accurately captures the true pattern of variation within the stalk, albeit with a lower spatial resolution.

### Other testing approaches

**Fig. 9:**
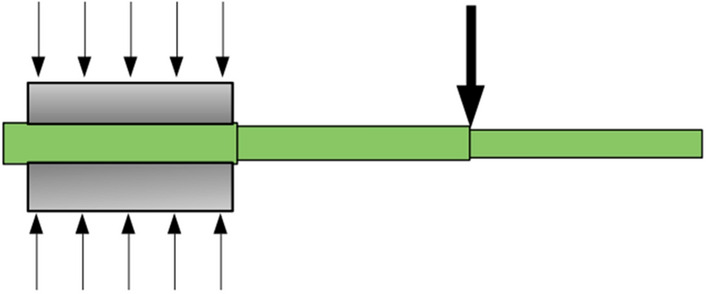
Schematic diagram of a cantilever testing arrangement

The astute reader may wonder why the authors did not use a cantilever testing approach such as one shown in Fig. 9. By clamping each internode in turn, this testing approach would provide one flexural stiffness value for each flexural test, with no ill-conditioning and spatial resolution at the level of the internode. Unfortunately the irregular shape of most plant stems makes this testing approach impractical. Clamps that are essentially rigid (steel, aluminum, etc.) will not conform to the plant stem, and thus allow some amount of bending deformation to the left of the cantilever point. This bending will adversely affect the measurements. If compliant (i.e. stiff foam or rubber) clamps are used, the compliance itself will also adversely affect the measurements. To be successful, this testing method would require a specially-designed clamp that can be articulated to precisely match the profile of the stem and then locked into place. Such a mechanism is certainly possible, but would require a significant amount of mechanical design work (Fig. 9).

Other approaches that were not investigated in this study include quantification of the displacement pattern along the length of the entire stalk. For example, optical methods of visualizing the displacement pattern of the entire stalk during bending tests have previously been used to assess bending displacement patterns [9, 20]. Such methods provide detailed deformation data with a single test. With further research, such methods may be amenable to solving for individual EI values. Alternatively, strain gauges may be used to complement the data of bending tests, thus potentially leading to new or modified methods that do not amplify experimental uncertainties.

### Limitations

One limitation of this study was the use of Euler–Bernoulli beam models for all results related to uncertainty. The Euler–Bernoulli model neglects shear deformation, which would be present to some degree in all actual physical tests, thus causing a minor discrepancy between these results and real physical tests. Shear deformation was neglected since the authors are not aware of any studies that have reported on the shear modulus of maize stalk tissues. In addition, the Timoshenko shear coefficient for maize stems is unknown. It is therefore difficult to quantify the effect of neglecting shear in this case. But, shear deformation is very minor for most flexural tests performed on plant stems [18]. Further research would be needed to quantify this effect more adequately.

A second limitation is the assumption of uniform flexural stiffness for each nodal segment. While this first-order approximation has been used in this and previous studies [7], it neglects the influence of nodal regions. Data on the profile of morphology of maize stalks (Fig. 1) [10] suggest that nodes are stiffer than both neighboring internodes. However, at least one previous study suggested that nodes are less stiff than internodes, with internodes being more rigid [20]. Further research is therefore needed to confirm the influence of nodes on the overall flexural stiffness of plant stems. Alternatively, it may be insightful to model the maize stalk as a continuously tapering beam, as has been done in other studies of leaf petioles and feathers [21–23].

Finally, in this study the system of equations and uncertainty characteristics were outlined for a stem consisting of 4 internode (5 nodes). The method can certainly be extended to longer stems consisting of more internodes, but this was not undertaken in this study. One challenge in doing so is that the number of possible test combinations increases rapidly as additional internodes are added. For example, 20 unique tests are possible for a stem having 5 internodes, with over 1 million possible test combinations.

## Conclusions

We have demonstrated that the flexural stiffness of individual internodes can be determined using the methods described in this paper. The individual internode method involves multiple non-destructive three-point bending tests. Test results are then used in a linear system of equations to obtain the flexural stiffness of individual internode segments. Multiple combinations of tests are possible, but all combinations were found to be ill-conditioned. As a result, errors in the measurement of length and force/displacement slope are amplified, resulting in outputs (results) that exhibit significantly higher errors than the constituent inputs. An uncertainty analysis was carried out to investigate possible means of minimizing errors. We found that certain combinations of physical tests minimize uncertainty in the solution, and that row-wise scaling reduced the level of error. However, even the best test combinations amplify measurement error by a factor of at least 6. Careful consideration should therefore be paid to measurement errors. If measurement errors exceed the average difference in flexural stiffness between adjacent segments, patterns of flexural stiffness may be obscured or distorted. An alternative to the individual method is the aggregate method. This method determines the aggregate flexural stiffness of segments consisting of two adjacent internodes. This method requires fewer tests and does not amplify measurement error. The drawback to this method is that it provides lower spatial resolution than the individual method.

Relationships between input and output errors were described for both methods. This information indicates that sufficiently accurate measurement of the internode lengths and the force/displacement slope of flexural tests can be used to obtain meaningful results. Future researchers can use the information in this paper to make an informed decision about which testing method is more suitable, given a particular set of measurement equipment, time constraints, and other factors.

## Data Availability

The datasets during and/or analysed during the current study available from the corresponding author on reasonable request.

## Abbreviations

*A*, *B*, *C*, *D*: Individual internodes
*a*, *b*, *c*, *d*: Lengths of internodes
*K*: Flexural stiffness. *K*_*A*_ indicates the flexural stiffness of internode *A*. *K*_*AB*_ indicates the aggregate flexural stiffness of the stalk segment *AB*
F: Force
*δ*: Displacement
*E*: Young’s Modulus
*I*: Area moment of inertia
*L*: Length
*ɸ*: The slope of the linear segment of a force/displacement curve
*u*: Standard uncertainty (standard deviation of the errors)
*u*_95_: The half-width of 95% confidence interval
*ϵ*: Error, the difference between true and measured values
